# Reversible Blockade of Complex I or Inhibition of PKCβ Reduces Activation and Mitochondria Translocation of p66^Shc^ to Preserve Cardiac Function after Ischemia

**DOI:** 10.1371/journal.pone.0113534

**Published:** 2014-12-01

**Authors:** Meiying Yang, David F. Stowe, Kenechukwu B. Udoh, James S. Heisner, Amadou K. S. Camara

**Affiliations:** 1 Department of Anesthesiology, Medical College of Wisconsin, Milwaukee, WI, United States of America; 2 Department of Physiology, Medical College of Wisconsin, Milwaukee, WI, United States of America; 3 Cardiovascular Research Center, Medical College of Wisconsin, Milwaukee, WI, United States of America; 4 Research Service, Zablocki VA Medical Center, Milwaukee, WI, United States of America; 5 Department of Biomedical Engineering, Marquette University, Milwaukee, WI, United States of America; University of Cincinnati, College of Medicine, United States of America

## Abstract

**Aim:**

Excess mitochondrial reactive oxygen species (mROS) play a vital role in cardiac ischemia reperfusion (IR) injury. P66^Shc^, a splice variant of the ShcA adaptor protein family, enhances mROS production by oxidizing reduced cytochrome *c* to yield H_2_O_2_. Ablation of p66^Shc^ protects against IR injury, but it is unknown if and when p66^Shc^ is activated during cardiac ischemia and/or reperfusion and if attenuating complex I electron transfer or deactivating PKCβ alters p66^Shc^ activation during IR is associated with cardioprotection.

**Methods:**

Isolated guinea pig hearts were perfused and subjected to increasing periods of ischemia and reperfusion with or without amobarbital, a complex I blocker, or hispidin, a PKCβ inhibitor. Phosphorylation of p66^Shc^ at serine 36 and levels of p66^Shc^ in mitochondria and cytosol were measured. Cardiac functional variables and redox states were monitored online before, during and after ischemia. Infarct size was assessed in some hearts after 120 min reperfusion.

**Results:**

Phosphorylation of p66^Shc^ and its translocation into mitochondria increased during reperfusion after 20 and 30 min ischemia, but not during ischemia only, or during 5 or 10 min ischemia followed by 20 min reperfusion. Correspondingly, cytosolic p66^Shc^ levels decreased during these ischemia and reperfusion periods. Amobarbital or hispidin reduced phosphorylation of p66^Shc^ and its mitochondrial translocation induced by 30 min ischemia and 20 min reperfusion. Decreased phosphorylation of p66^Shc^ by amobarbital or hispidin led to better functional recovery and less infarction during reperfusion.

**Conclusion:**

Our results show that IR activates p66^Shc^ and that reversible blockade of electron transfer from complex I, or inhibition of PKCβ activation, decreases p66^Shc^ activation and translocation and reduces IR damage. These observations support a novel potential therapeutic intervention against cardiac IR injury.

## Introduction

Mitochondria are proximal effectors and determinants of cell fate during ischemia and reperfusion (IR)-mediated oxidative stress. Thus they are also potential therapeutic targets to ameliorate oxidative damage [1]. Excess mitochondrial reactive oxygen species (mROS) emission plays a key role in contributing to cardiac IR injury [2]. It is generally accepted that in mitochondria the superoxide anion (O_2_^−•^), the precursor of most ROS, is generated within the electron transport chain (ETC) complexes (e.g. I, II and III), wherein the leak of a single electron reduces O_2_ to O_2_^−•^ [3]–[5].

Recent reports indicate that p66^Shc^, a splice variant of the ShcA adaptor protein family, also contributes to mROS production [1], [6]. Giorgio et al. [6] suggested that p66^Shc^ utilizes reducing equivalents of the ETC by oxidizing reduced cytochrome *c* (cyt *c*) to catalyze the reduction of O_2_ to H_2_O_2_. Electron transfer from cyt *c* to p66^Shc^ would designate it as a mitochondrial redox enzyme [6] that could play an alternative role as a signaling molecule for mitochondrial-mediated cell apoptosis [7], [8]. Indeed, p66^Shc^ gene ablation (p66^Shc−/−^) has been shown to reduce hypoxia/reoxygenation-induced damage to hepatocytes [9] and to decrease necrosis and apoptosis of myofibrils after hind limb ischemia compared to the wild type [10]. Furthermore, in isolated perfused hearts, p66^Shc−/−^ mice compared to wild type mice exhibited both reduced IR-mediated LDH release into the coronary effluent and abrogated lipoperoxidation [11].

The pathway leading to p66^Shc^ activation and translocation into mitochondria is unclear. Excess H_2_O_2_ or ultraviolet light (UV) irradiation has been shown to activate a serine-threonine protein kinase C β (PKCβ), which led to p66^Shc^ phosphorylation at serine 36, and to trigger mitochondrial accumulation of the protein after its recognition by the prolyl isomerase Pin1 in mouse embryonic fibroblasts (MEF) [12], [13]. Pinton et al. [13] reported that in MEF, inhibition of PKCβ with hispidin inhibited H_2_O_2_ -induced p66^Shc^ phosphorylation; overexpression of PKCβ mediated H_2_O_2_-induced mitochondrial dysfunction in wild type MEFs, but not in p66^Shc−/−^ MEFs. It was reported that activation of PKCβII in ventricular tissue increased after IR and that gene deletion or pharmacological blockade of PKCβII was associated with protection against ischemia [14].

Mitochondrial ETC complexes are involved in mROS production during IR. Moreover, O_2_^−•^ generated at mitochondrial complex III can be attenuated by limiting electron transfer from complex I, thereby provide protection against IR injury. We [15], and others [16], have reported that the therapeutic targeting of complex I with amobarbital provided cardioprotection, in part, by decreasing mROS production during IR. Amobarbital, a short-acting barbiturate, reversibly attenuates complex I electron transfer at the rotenone site [17], decreased IR-induced O_2_^−•^ generation and mitochondrial [Ca^2+^] overload [15], retarded mitochondrial permeability transition pore (mPTP) opening [16], and improved oxidative phosphorylation (OxPhos) [16]. These mitochondrial effects culminated in appreciable protection of cardiac function on reperfusion after ischemia [15], [16]. However, targeting distal complexes of the ETC, especially complex IV, is not protective against ischemic stress and may exacerbate injury. For example, blocking complex IV before ischemia increased levels of reduced cyt *c*, a likely substrate for p66^Shc^-mediated H_2_O_2_ generation [6] leading to more oxidative stress.

Our aims were to explore if p66^Shc^ is involved in IR induced mROS generation and how ROS and p66^Shc^ dynamically modulate each other during different periods of cardiac ischemia and reperfusion. To address these objectives, we used the perfused *ex vivo* guinea pig heart model and monitored: a) if and when p66^Shc^ is activated during cardiac ischemia and/or reperfusion; b) if activation of PKCβII during IR induces p66^Shc^ activation and mitochondrial translocation to contribute to cardiac IR injury; c) if reversible attenuation of complex I electron transfer with amobarbital during IR is associated with p66^Shc^ activation.

## Methods

### Ethics Statements

Our animal protocols conformed to the Guide for the Care and Use of Laboratory Animals (National Institutes of Health No. 85-23, Revised 1996). The Medical College of Wisconsin IACUC, with the number AUA 1647, approved all our animal studies.

### Isolated heart preparation and measurements

Hearts were removed and prepared for study as described previously [18]–[22]. In brief, guinea pigs were given heparin (1000 units) and ketamine (50 mg/kg) i.p. before sacrifice. Hearts were harvested and perfused retrograde at constant pressure (55 mmHg) via the aortic root with an oxygenated Krebs-Ringer's (KR) solution of the following composition (in mM): 138 Na^+^, 4.5 K^+^, 1.2 Mg^2+^, 2.5 Ca^2+^, 134 Cl^−^, 15 HCO_3_^−^, 1.2 H_2_PO_4_^−^, 11.5 glucose, 2 pyruvate, 16 mannitol, 0.1 probenecid, 0.05 EDTA, and 5 U/L insulin and gassed with 3% CO_2_, 97% O_2_ (pH 7.4) at 37°C. Systolic and diastolic left ventricular pressures (LVP), coronary flow (CF) and heart rate were measured online continuously for all of perfused hearts as described previously [18]–[22]. The aortic inflow line was clamped to induce global ischemia. If ventricular fibrillation (VF) occurred on reperfusion, 250 µg of lidocaine was given via the aortic cannula to restore sinus rhythm.

### Protocols

At the end of the indicated times of ischemia and/or reperfusion, some hearts were removed and immediately snap frozen in liquid N_2_ and stored at −80°C for later measurement of p66^Shc^ and PKCβII phosphorylation (n = 3 hearts per group). Other hearts were removed and mitochondria were immediately isolated for assessment of cytosolic and mitochondrial changes in p66^Shc^ levels (n = 3 hearts per group). Other hearts were removed at 120 min reperfusion to measure infarct size (n = 8 hearts per group). In the complex I blocker treated groups, hearts were perfused with 2.5 mM amobarbital (Amo) for 1 min before initiating global ischemia [15]; in the PKCβ antagonist treated groups, hearts were perfused with 40 µM hispidin (His) for 10 min before ischemia. Amobarbital and hispidin were administered up to the initiation of ischemia to ensure the presence of the drugs in the heart during the entire period of ischemia and briefly during the onset of reperfusion.

### Infarct size measurement

After 120 min reperfusion, hearts were removed and the atria discarded. The ventricles were cut into 3 mm sections, and then stained with 1% 2,3,5-triphenyltetrazolium chloride (TTC) to measure infarct size. In living tissue, TTC is reduced by pyridine nucleotide-linked dehydrogenase into a red, lipid-soluble formazan that stains living tissue red. Infarcted tissue lacking this dehydrogenase remains unstained. Infarct size of the TTC stained white area was determined as a percentage of total ventricular heart weight [22], [23].

### Measurement of NADH in isolated hearts

Tissue autofluorescence (arbitrary fluorescence units, afu) is an indicator of NADH at λ_em_ 460 nm (λ_ex_ 350 nm); it is primarily utilized to assess the mitochondrial redox state (n = 6 isolated hearts/group) [15], [24], [25]. Motion artifact was reduced by using λ_em_ 405 nm as a reference that is less sensitive to changes in NADH [26]. Therefore, the ratio of auf at λ_em_ 460/λ_em_ 405 nm indicates mitochondrial NADH.

### Detection of O_2_^−•^ in isolated hearts loaded with dihydroethidium (DHE)

The intracellular fluorescent probe DHE (Molecular Probes) was used to assess O_2_^−•^ emission continuously during IR (n = 6 isolated hearts per group) as described previously [15], [20], [24], [27]. After stabilization, hearts were loaded with 10 µM DHE dissolved in KR solution for 20 min; this was followed by washout of residual, unincorporated DHE with KR solution for 20 min [15], [20]. The fluorescence emitted (afu) after washout was adjusted to 0 afu (baseline) to normalize the fluorescence intensity for all experiments. Changes in the DHE fluorescence signal, which represents O_2_^−•^ generation, were compared to the baseline fluorescence signal values [15], [18], [27].

### Preparation of cytosolic and mitochondrial fractions

Mitochondrial and cytosolic fractions were prepared using procedures described previously [27]–[32] with minor modifications. All procedures were carried out at 4°C. Hearts were minced in a chilled isolation buffer containing (in mM) 200 mannitol, 50 sucrose, 5 KH_2_PO_4_, 5 MOPS, 1 EGTA and 0.1% fatty acid free BSA at pH 7.15, then homogenized. The homogenized slurries were centrifuged at 8000 *g* for 10 min. The supernatant were collected and further centrifuged at 50,000 *g* for 30 min, and then the resulting supernatant was used as the cytosolic fraction. The pellet from the 8,000 *g* centrifugation was resuspended in isolation buffer containing protease inhibitors and spun at 750 *g* for 10 min; the supernatant was collected and again centrifuged at 8000 *g*. The final mitochondrial pellet following this centrifugation was resuspended in isolation buffer and then purified as described by Graham [33]. Mitochondria were layered on 30% Percoll in isolation buffer, and then centrifuged for 30 min at 95,000 *g*. After centrifugation, the lower part of the dense brown band containing the purified mitochondria was collected and washed two times with isolation buffer. After purification, the mitochondria were resuspended in isolation buffer containing protease inhibitors and stored at −80°C for later use. Mitochondrial and cytosolic protein concentrations were assessed using Bio-Rad protein assay with bovine serum albumin (BSA) as the standard.

### Immunoprecipitation of mitochondrial proteins

Immunoprecipitation (IP) was performed as described previously [27], [34], [35] with minor changes. Frozen hearts were pulverized under liquid nitrogen and then the powder was lysed in RIPA buffer containing 50 mM Tris-Cl pH 7.4, 150 mM NaCl, 1% deoxycholate, 1% Triton X-100, 0.1% SDS and protease inhibitors on ice for 30 min. The sample was pre-cleared with 30 µl protein G Sepharose-4B beads (Invitrogen) for 1 h at 4°C with constant end-over-end shaking and then centrifuged at 3000 *g* for 5 min. Supernatant was collected, adjusted to 2 mg/ml protein concentration with RIPA buffer containing protease inhibitors, and subjected to IP with an anti-Shc antibody (rabbit polyclonal IgG, Millipore) at 4°C with constant end-over-end shaking overnight. The next day 30 µl of protein G Sepharose-4B beads was added and the mixture was incubated for another 2 h under the same conditions as above. The beads were collected and washed (five times) with RIPA buffer. After washing and aspirating the washing buffer completely, 50 µl Laemmli sample buffer containing 50 mM Tris-Cl, 10% glycerol, 500 mM β-mercaptoethanol, 2% SDS, 0.01% w/v bromophenol blue and protease inhibitors at pH 7.4, was added to the beads and boiled at 95°C for 5 min. The immunoprecipated proteins were separated using SDS-PAGE and then subjected to Western blot analyses.

### Western blot analyses

Mitochondrial protein (50 µg) or immunoprecipitates were resolved by SDS-PAGE and transferred onto a PVDF membrane. Membranes were incubated with specific primary antibodies: anti-Shc/p66 (pSer36) (mouse monoclonal, 1∶1000, Calbiochem); anti-Shc (total pool of p66^Shc^, mouse monoclonal, 1∶1000, BD Transduction Laboratories); PKCβII (rabbit polyclonal, 1∶500 Santa Cruz); and P-PKCβII/δ (Ser660) (rabbit polyclonal, 1∶500, Santa Cruz). Washed membranes were incubated with the appropriate secondary antibody conjugated to HRP, then immersed in an enhanced chemiluminescence detection solution (GE Healthcare) and exposed to X-Ray film for autoradiography. To monitor the amounts of loaded proteins and purity of isolated mitochondrial and cytosolic fractions, specific antibody-blotted membranes were stripped and then probed with antibodies against VDAC (rabbit polyclonal, 1∶1000, Cell Signaling) or β-actin (rabbit polyclonal, 1∶500, Santa Cruz).

### Statistical analysis

All results were expressed as means ±SEM and were analyzed by one-way ANOVA followed by a post-hoc analysis (Student-Newman-Keuls' test) to determine significant differences of means among groups. Isolated heart data were collected for statistical evaluation at time points 30, 45, 50, and 65 min. *P*<0.05 was considered significantly different (two-tailed).

## Results

### Activation of p66^Shc^ occurred during reperfusion after ischemia

**Figure 1:**
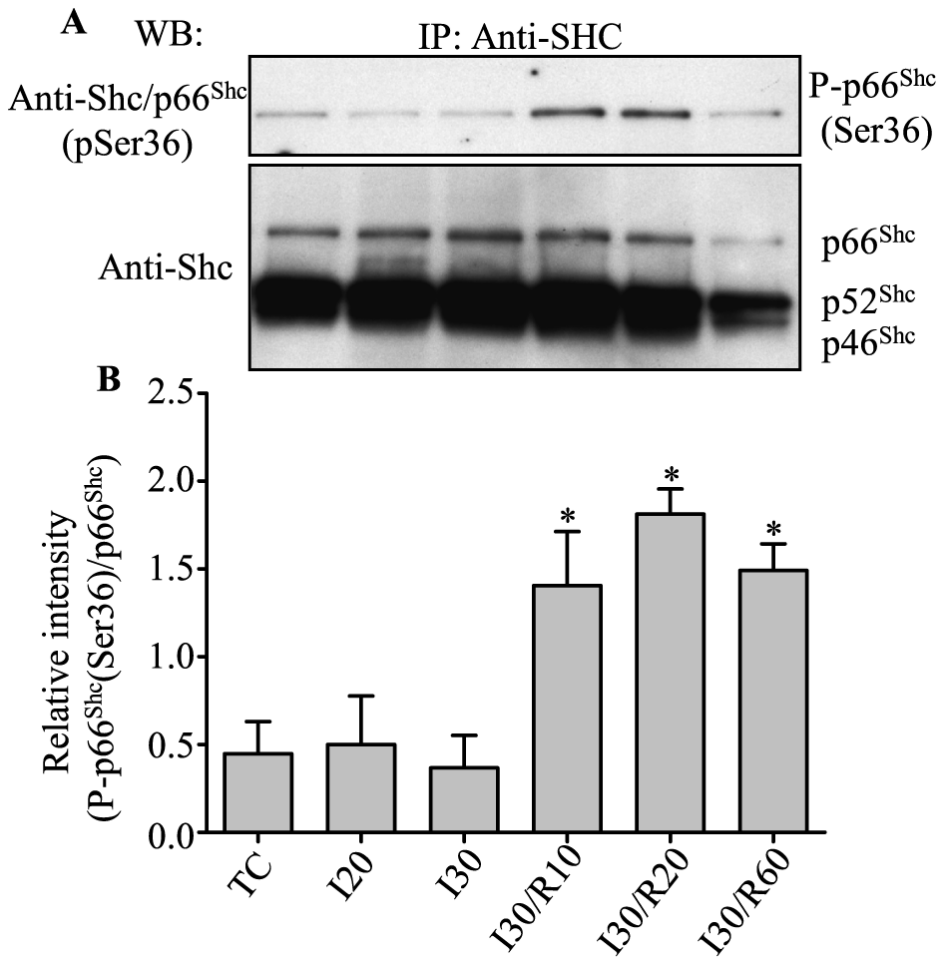
Phosphorylation of p66^Shc^ at Ser36 increased during reperfusion (R) after ischemia (I) in isolated guinea pig hearts compared to no ischemia (time controls, TC). **A**, upper panel: phosphorylation of p66^Shc^ at Ser36 from total protein lysate of heart tissue. **A**, lower panel: total p66^Shc^ level for loading control. Summary of mean band intensities (**B**) derived from three independent experiments (n = 3 hearts/group); band intensities were determined by densitometry using imageJ software and the intensities of P-p66 (Ser36) were normalized to the total p66^Shc^ in each sample. *P*<0.05: *IR *vs.* TC and I.

We first examined if and when p66^Shc^ was activated during cardiac IR by determining the level of phosphorylation of p66^Shc^ at serine 36 (Ser36), an indication of p66^Shc^ activation [12], [13]. After normalizing to total immunoprecipated p66^Shc^ (Fig. 1A, bottom panel), phosphorylation of p66^Shc^ at Ser36 increased on reperfusion in the IR groups compared to the time control (TC) and ischemia only groups (Fig. 1A, upper panel); the increase in phosphorylation of p66^Shc^ at Ser36 occurred as early as 10 min reperfusion after 30 min ischemia and was detectable for up to 60 min reperfusion. In the 20 and 30 min ischemia only groups, the phosphorylation of p66^Shc^ at Ser36 did not change compared to the TC group. These results indicated that IR induced p66^Shc^ activation and, moreover, that p66^Shc^ was activated only during reperfusion after ischemia and not by ischemia alone.

### P66^Shc^ localized to mitochondria during reperfusion after ischemia

**Figure 2:**
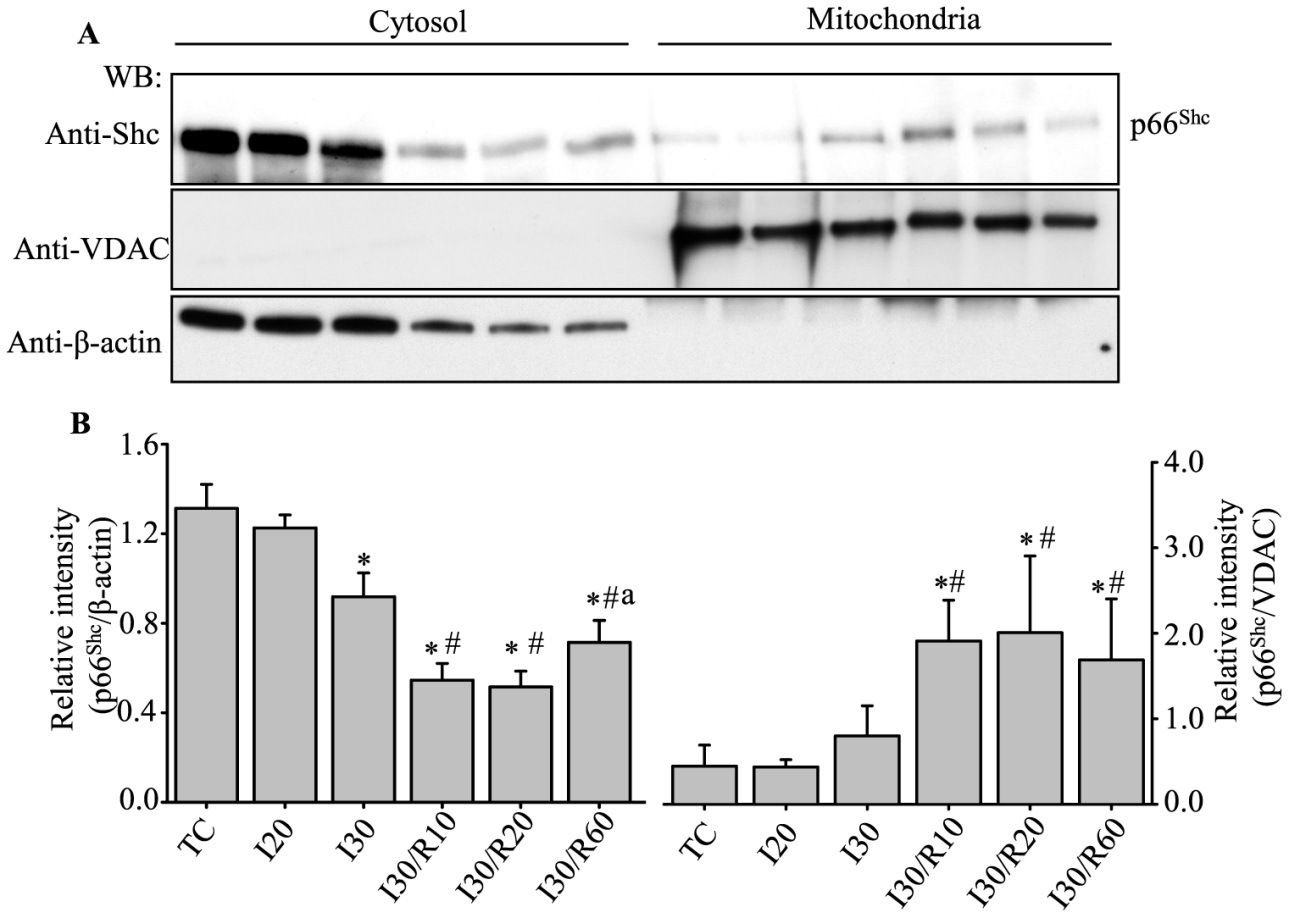
P66^Shc^ expression levels changed in cytosol and mitochondria during reperfusion after ischemia. **A**, upper panel: p66^Shc^ level in mitochondrial and cytosolic fractions. Anti-VDAC (**A**, middle panel) and anti-β–actin (**A**, lower panel) confirm protein loading, mitochondrial (β–actin) and cytosolic purity (VDAC). Summary of mean band intensities (**B**) derived from three independent experiments (n = 3 hearts/group) (see Fig. 1 for imaging). *P*<0.05: *IR *vs.* TC and I20; # IR *vs* I30; ^a^ I30R60 *vs* I30R10 or I30R20.

It was reported that after phosphorylation at Ser36, p66^Shc^ is recognized by prolyl isomerase Pin1, which enables the phosphorylated p66^Shc^ to be translocated to the IMM [13]. We therefore examined the translocation of p66^Shc^ into mitochondria during cardiac IR injury. At the end of the indicated ischemia and/or reperfusion period, hearts were removed and cytosolic and mitochondrial fractions were prepared (see Methods), and then subjected to Western blot analyses with anti-Shc antibody. The level of p66^Shc^ in mitochondria increased by approximately 2-fold at 10 and 60 min reperfusion, and by approximately 3-fold at 20 min reperfusion after 30 min ischemia, compared to TC (Fig. 2). The level of p66^Shc^ in the cytosolic fraction decreased in the 30 min ischemia alone group and in the 30 min ischemia followed by 10, 20 or 60 min reperfusion groups when compared to the TC and 20 min ischemia groups (Fig. 2).

The incongruity in the reverse changes in p66^Shc^ levels in cytosolic and mitochondrial fractions in the 30 min ischemia alone group indicated that p66^Shc^ might translocate to other parts of the cell, including organelles other than mitochondria. Moreover, the cytosolic p66^Shc^ level was lower in the 30 min ischemia plus 10 and 20 of reperfusion groups when compared to the 30 min ischemia plus 60 min of reperfusion group (Fig. 2). These results suggested greater cell injury/death after 60 min of reperfusion contributed to the lower p66^Shc^ levels. The translocation of p66^Shc^ into mitochondria during reperfusion after 30 min ischemia was consistent with phosphorylation of p66^Shc^ at Ser36 during the indicated periods (Fig. 1). Insofar as ischemia alone did not lead to phosphorylation of p66^Shc^, correspondingly, ischemia alone (20 or 30 min) also did not show significant mitochondrial translocation of p66^Shc^ when compared to their respective TCs.

### Prolonged ischemia required to activate p66^Shc^ during reperfusion after ischemia

**Figure 3:**
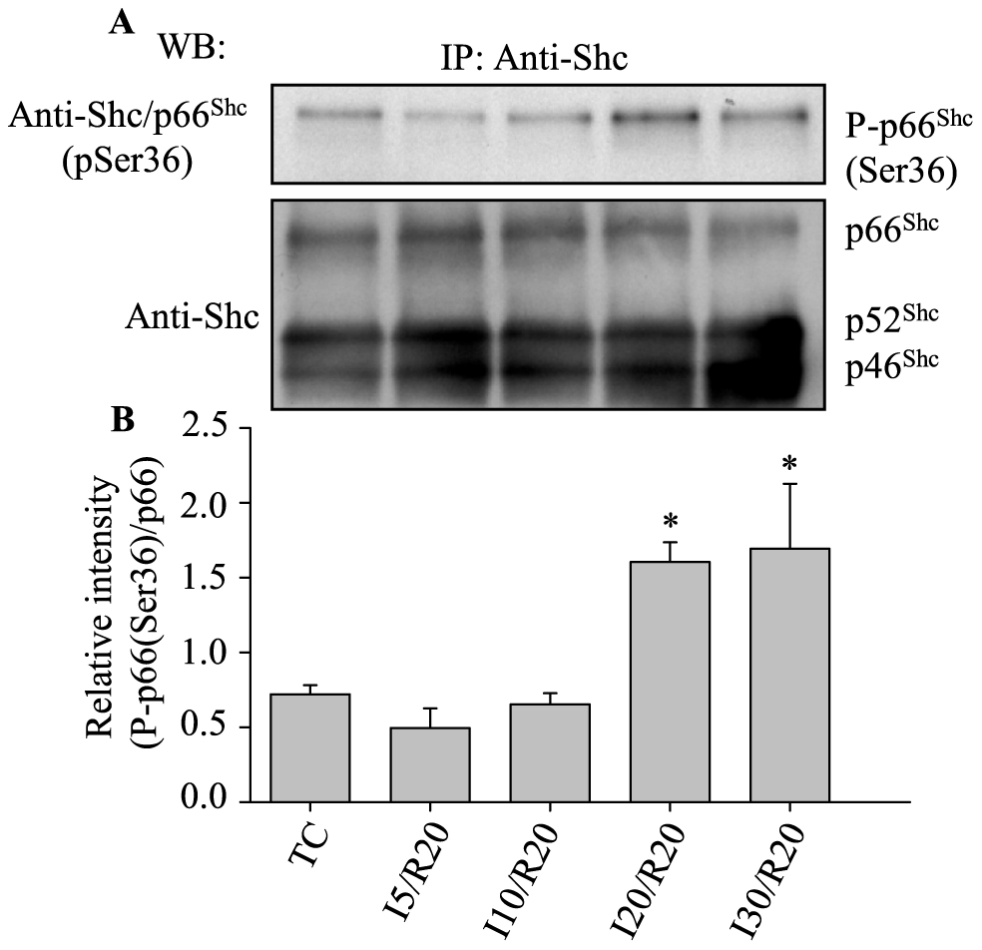
Phosphorylation of p66^Shc^ at Ser36 during reperfusion after ischemia was dependent on the duration of ischemia. **A**, upper panel: phosphorylation of p66^Shc^ at Ser36; **A**, lower panel: total p66^Shc^ level for loading control. **B**: Summary of mean band intensities derived from three independent experiments (n = 3 hearts/group) (see Fig. 1 for imaging). *P*<0.05: *I20 and I30 *vs.* I5, I10 and TC.

Our previous reports show that the magnitude of ROS production during ischemia is dependent on the duration of ischemia [15], [20], [27]. Next, we investigated if the activation of p66^Shc^ was associated with the duration of ischemia. Guinea pig isolated hearts were subjected to 5, 10, 20 or 30 min ischemia, followed by 20 min reperfusion. At the end of reperfusion, hearts were harvested and evaluated for phosphorylation of p66^Shc^ at Ser36. Phosphorylation of p66^Shc^ at Ser36 increased after 20 min reperfusion following 20 or 30 min global ischemia, but not after 5 or 10 min ischemia plus 20 min reperfusion compared to their respective TCs (Fig. 3). These results indicated that at least 20 min ischemia was required for some p66^Shc^ activation during reperfusion after ischemia.

**Figure 4:**
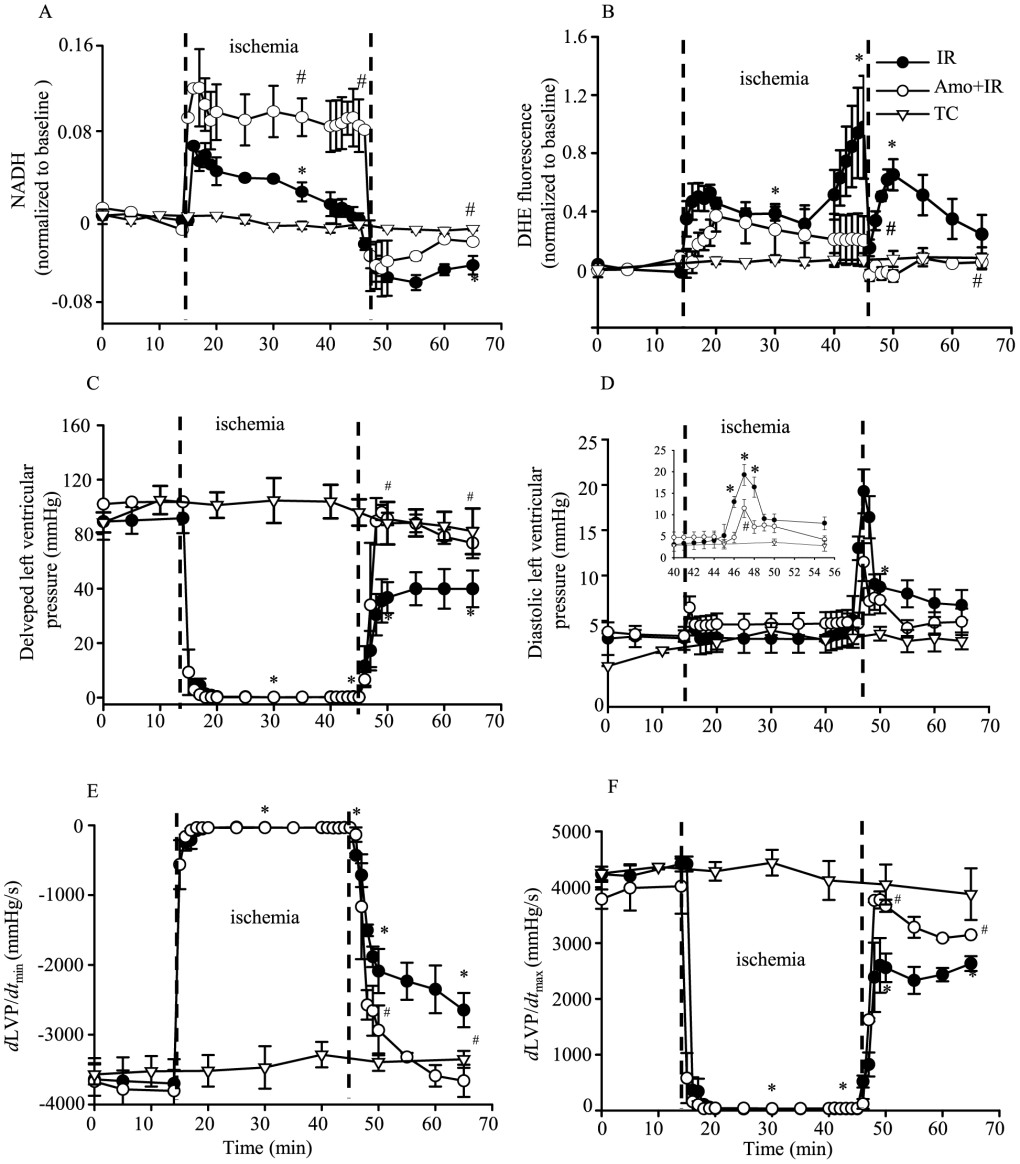
NADH autofluorescence (afu) (A) was higher and DHE fluorescence intensity (O_2_^−•^ emission) (B) was lower during ischemia and reperfusion after amobarbital (Amo) treatment compared to IR alone. DHE and NADH were recorded online in isolated hearts at the LV free wall. n = 6 hearts/group/fluorescence measure. Cardiac contractile and relaxant function: developed LVP (**C**), diastolic LVP (**D**), *d*LVP/*dt*_min_ (**E**) and *d*LVP/*dt*_max_ (**F**) were improved on reperfusion after amobarbital (Amo) treatment during ischemia. Inset in **D** shows detailed changes in diastolic LVP during late ischemia and early reperfusion. n = 9 hearts/group/functional measure. All values are means±SEM. TC, time control; IR, ischemia and reperfusion. *P*<0.05: *IR *vs.* TC; ^#^Amo+IR *vs.* IR.

To evaluate a possible correlation between p66^Shc^ activation and translocation with the mitochondrial redox state and generation of ROS, online mitochondrial NADH and DHE (O_2_^−•^ emission) fluorescence intensities were measured during 30 min ischemia and 20 min reperfusion. During early ischemia NADH increased markedly, but then gradually declined toward baseline levels as ischemia progressed (Fig. 4A, IR); during reperfusion, NADH was lower than baseline (Fig. 4A, IR). These findings indicated a shift in mitochondrial redox towards more oxidized mitochondria during early reperfusion. The corresponding time-dependent changes in DHE fluorescence signals were characterized by a modest steady increase during early ischemia and a marked and accelerated increase during late ischemia (Fig. 4B, IR); the DHE fluorescence signal intensity remained elevated above baseline during reperfusion in the IR group.

### Amobarbital decreased p66^Shc^ activation induced by ischemia and reperfusion

**Figure 5:**
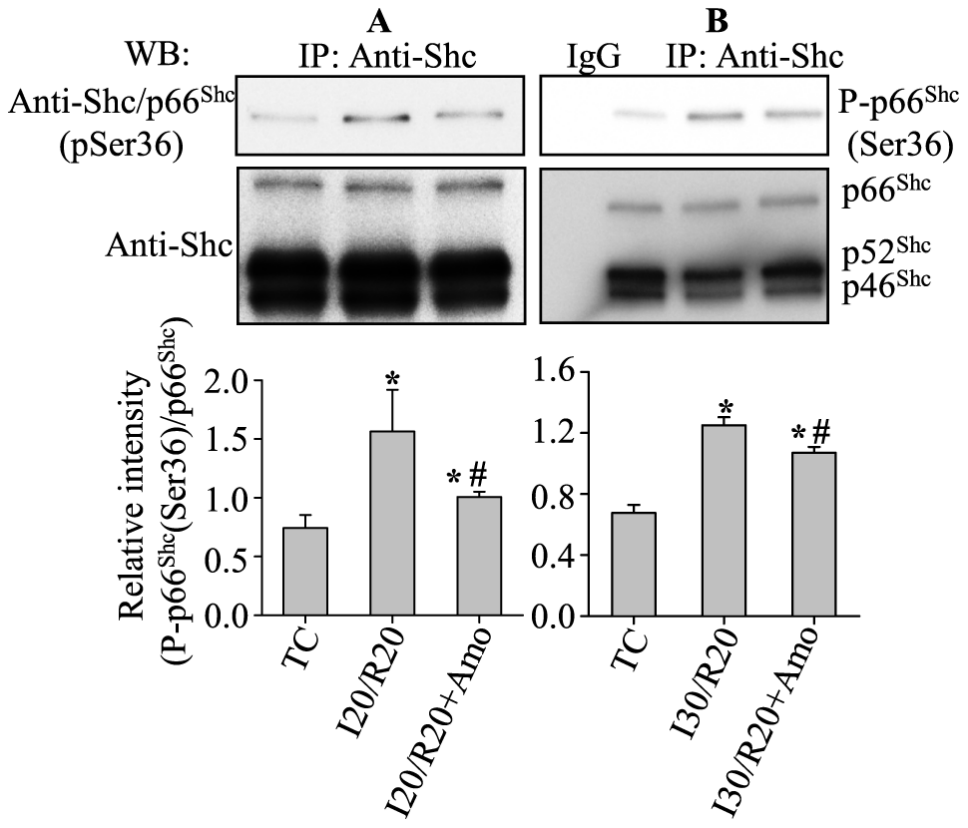
IR-induced phosphorylation (P) of p66^Shc^ at Ser36 was decreased by amobarbital (Amo) after 20 (A) or 30 (B) min ischemia (I) followed by 20 min reperfusion (R). **A**, **B**, upper panel: phosphorylation of p66^Shc^ at Ser36; equal amounts of p66^Shc^ loading were verified with anti-ShC antibody (**A**, **B**: middle panel). Summary of mean band intensities derived from three independent experiments (n = 3 hearts/group) (**A**, **B**: lower panel). IP, immunoprecipitation; TC, time controls (no IR). *P*<0.05: *IR *vs.* TC; ^#^Amo+IR *vs.* IR.

We next sought to determine if reversible blockade of complex I with amobarbital, which we showed previously reduced O_2_^−•^ emission during IR [15], would reduce p66^Shc^ activation during reperfusion. To achieve this, hearts were treated with or without amobarbital (Amo) prior to ischemia followed by 20 or 30 min ischemia and 20 min reperfusion. At the end of reperfusion, hearts were collected and evaluated for phosphorylation of p66^Shc^ at Ser36 (see Methods). Compared to the IR alone group, the amobarbital treated groups exhibited reduced phosphorylation of p66^Shc^ at Ser36 by 27.5±0.1% and 14.4±0.1% at 20 min reperfusion after 20 min (Fig. 5A) and 30 min (Fig. 5B) ischemia, respectively. However, phosphorylation of p66^Shc^ at Ser36 in the amobarbital treated groups was significantly higher than in the TC group. These data indicated, and supported our previous findings [15], that amobarbital-induced interference with mitochondrial electron transfer provides protection, at least in part, by attenuating p66^Shc^ activation during IR.

Therefore, to ascertain if attenuation of p66^Shc^ activation by amobarbital correlated with mitochondrial redox state, ROS production, and functional recovery during IR injury, we monitored NADH, O_2_^−•^ generation and several indices of contractility and relaxation online in amobarbital treated and untreated hearts during IR. Compared to untreated hearts, amobarbital treated hearts displayed better preserved mitochondrial NADH levels during 30 min ischemia and 20 min reperfusion (Fig. 4A; Amo+IR), and a decrease in DHE fluorescence (O_2_^−•^ production) during late ischemia (Fig. 4B; Amo+IR). Moreover, amobarbital treatment resulted in a significantly lower diastolic LVP during late ischemia and early reperfusion (Fig. 4D; Amo+IR) and improved developed LVP, *d*LVP/*dt*_min_ and *d*LVP/*dt*_max_ during early reperfusion (Fig. 4C, E, F; Amo+IR) compared to the IR alone group. The incidence of ventricular fibrillation (VF) in the IR untreated hearts was 100%, with an average of approximately 3 VF occurrences/heart during reperfusion; amobarbital treated hearts exhibited no incidence of VF during reperfusion. Each VF that occurred was subsequently reversed to sinus rhythm with lidocaine. It is noteworthy that although the 20 min ischemia plus 20 min reperfusion group demonstrated activation of p66^Shc^, there was no significant compromise in cardiac function or mitochondrial bioenergetics when compared to the TC group (data not shown). This suggested that p66^Shc^ activation is an early marker of ischemia.

### Activation of PKCβII induced by cardiac ischemia and reperfusion required for p66^Shc^ activation

**Figure 6:**
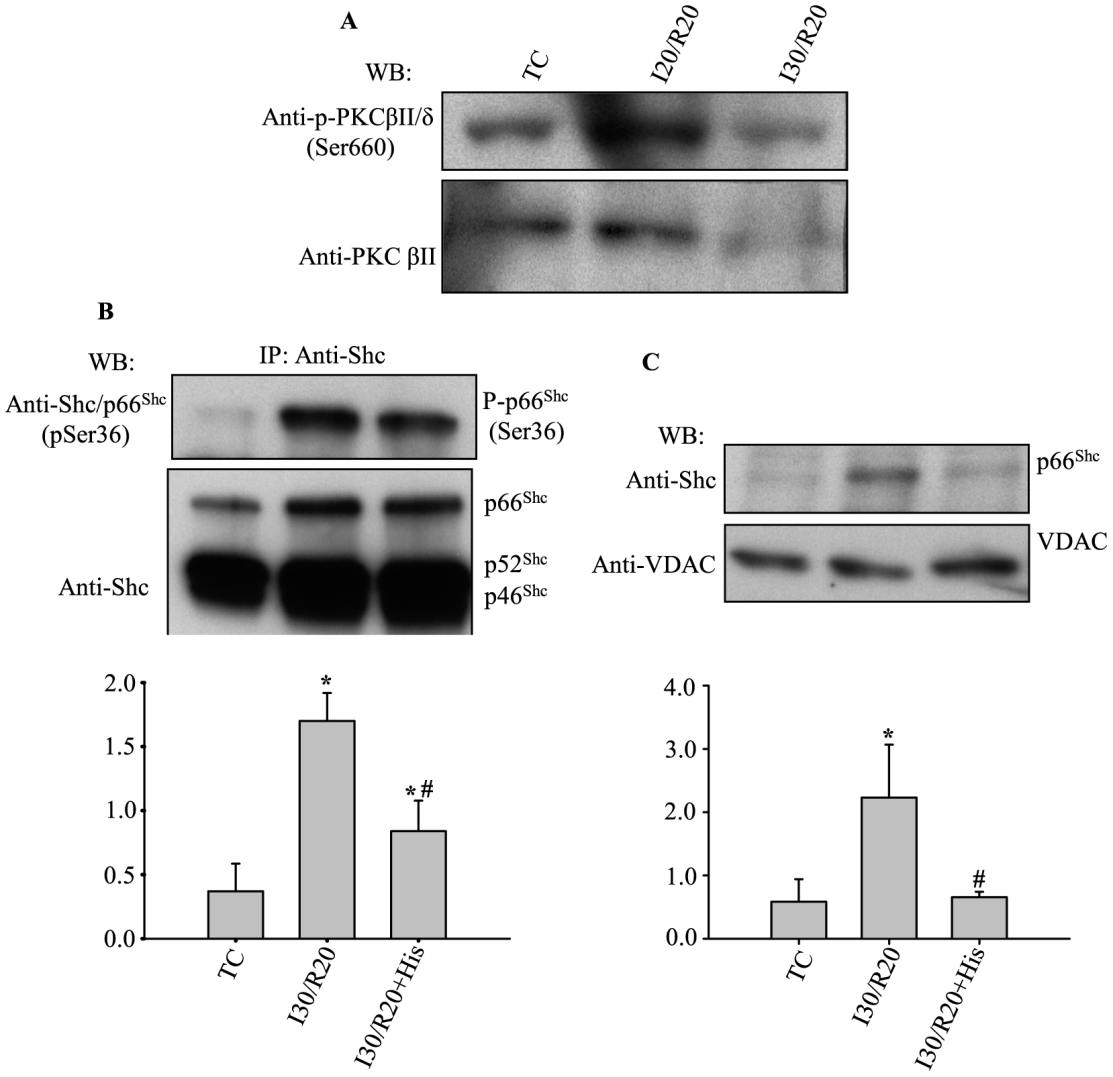
IR-induced activation of p66^Shc^ occurred via the PKCβII signaling pathway. **A**: Representative WB of 3 independent experiments of phosphorylation of PKCβII. **B**: Phosphorylation of p66^Shc^ at Ser36 during IR with or without hispidin (His) treatment. **C**: p66^Shc^ accumulation in mitochondria during IR with or without hispidin (His) treatment. **B, C** Lower panels show summary of mean band intensities derived from three independent experiments (n = 3 hearts/group) (see Fig. 1 for imaging). *P*<0.05: *IR *vs.* TC; ^#^IR+His *vs.* IR.

Next we examined if PKCβII is activated during cardiac IR, and if activation of p66^Shc^ during reperfusion after ischemia is mediated through PKCβII activated pathways as previously reported in MEFs [13], [36]. Western blot was used to determine phosphorylation of PKCβII at Ser660 in hearts subjected to 20 or 30 min ischemia plus 20 min reperfusion. Figure 6A shows that 20 or 30 min ischemia plus 20 min reperfusion increased phosphorylation of PKCβII at Ser660, suggesting IR activated PKCβII. Next we determined if PKCβII activation is required for p66^Shc^ activation during reperfusion. For this, hearts were treated with or without hispidin (His) for 10 min before the start of 30 min ischemia plus 20 min reperfusion. At the end of reperfusion, heart tissue was evaluated for phosphorylation of p66^Shc^ at Ser36; alternatively, mitochondria were isolated immediately after reperfusion to evaluate mitochondrial p66^Shc^ translocation. Figures 6B and C show that when hearts were perfused with hispidin before ischemia compared to IR only (control), both p66^Shc^ phosphorylation at Ser36 and p66^Shc^ mitochondrial translocation were decreased. These observations support the signaling role of PKCβ in activating the oxidoreductase, p66^Shc^, during oxidative stress in IR.

**Figure 7:**
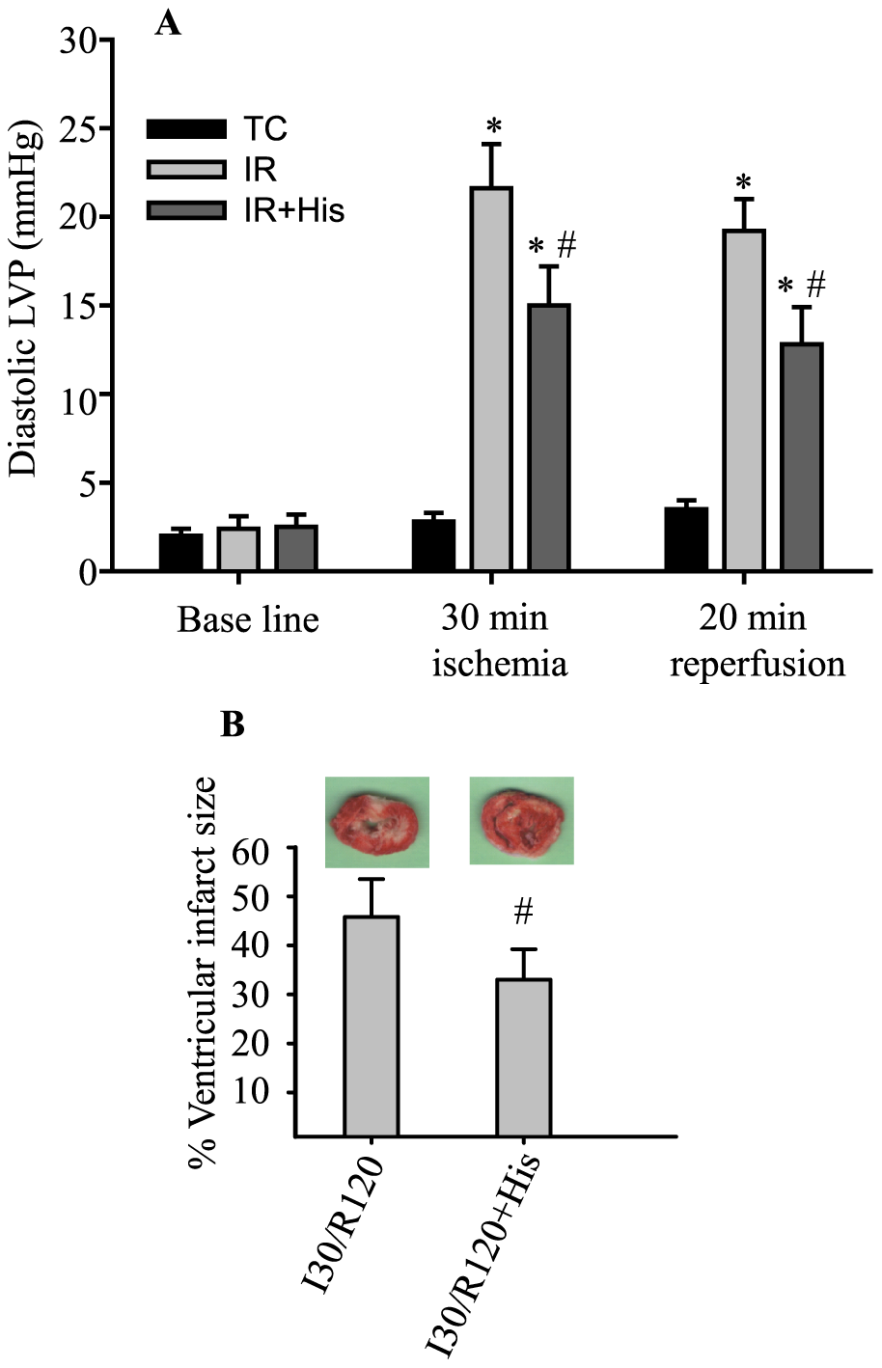
Inhibiting PKCβ activation reduced cardiac damage after IR. **A**: Diastolic LVP without IR (TC; n = 8 hearts/group) and before ischemia, at 30 min of ischemia and at 20 min of reperfusion (IR; n = 12 hearts/group) with or without hispidin (His; n = 10 hearts/group) treatment. **B**: Infarct size after 30 min of ischemia and 120 min of reperfusion (n = 8 hearts/group) with or without hispidin (His; n = 8 hearts/group) treatment. *P*<0.05: ^#^IR+His *vs.* IR.

We correlated attenuation of p66^Shc^ activation by hispidin with functional recovery and ventricular infarct size. Figure 7A shows that administration of hispidin before ischemia significantly reduced diastolic contracture, i.e. diastolic LVP, when compared to the IR only hearts. The incidence of VF after ischemia was significantly abated in hearts treated with hispidin before ischemia when compared to IR only hearts (data not shown). Figure 7B also shows that hispidin attenuated infarct size to 33±3% compared to the untreated IR (control) group in which infarct size was 45±3%. These results showed that ischemia followed by reperfusion induces p66^Shc^ activation through the PKCβII signaling pathway to contribute to IR injury.

## Discussion

We have demonstrated that p66^Shc^ is activated and translocated into mitochondria during global no-flow IR in the *ex vivo* perfused heart model. Activation of p66^Shc^ and its subsequent translocation into mitochondria are dependent both on the duration of ischemia and on the occurrence and timing of reperfusion after ischemia. Reversible attenuation of respiration via complex I by amobarbital reduced both ROS emission and activation of p66^Shc^ and improved function on reperfusion. Inhibiting PKCβ during IR with hispidin reduced activation/translocation of p66^Shc^, improved cardiac function, and decreased cell injury. Thus, p66^Shc^ is both a marker and a mitochondrial effector of oxidative stress-mediated mitochondrial dysfunction and ROS production. The effects of amobarbital on reducing mROS emission, improving redox state, and attenuating p66^Shc^ activation during IR injury, suggest an important link between oxidative stress and activation of p66^Shc^.

### P66^Shc^ is activated during reperfusion after global cardiac ischemia

IR injury can occur under a number of situations including cardiac arrest and resuscitation, hypoxia and reoxygenation, and coronary artery occlusion and reperfusion. Although ischemia itself can induce cardiac injury, much of the injury occurs during early reperfusion [37]. The causative and interactive factors of myocardial reperfusion injury include excess ROS, Ca^2+^ overload, and induction of apoptosis [38]. It is well known that the major source of ROS emission during reperfusion in cardiomyocytes is the ETC, which is progressively damaged during ischemia and contributes to further ROS generation during reperfusion [2], [20], [39]–[41].

However recent studies showed that p66^Shc^ may also act as an oxidoreductase to generate H_2_O_2_ in mitochondria after activation following redox stimulation [6]. Furthermore, in the mouse isolated heart model of IR injury, p66^Shc−/−^ mice showed less susceptibility to IR injury than wild type mice [11]. In the present study, we showed that p66^Shc^ only becomes activated and translocated to mitochondria during reperfusion after 20 or 30 min ischemia (Figs. 1, 2 and 3). This suggests that p66^Shc^ could also contribute to mROS generation during IR injury especially during reperfusion after ischemia. Why p66^Shc^ is activated only during reperfusion is unclear, but it is possible that the preceding events during ischemia (e.g. excess ROS emission) followed by restoration of flow, O_2_ and substrate supply, and by the rapid re-establishment of a reduced redox state and OxPhos, may collectively contribute to p66^Shc^ activation.

Ischemia, when prolonged, irreversibly disrupts electron transfer during reperfusion, yet ETC complexes I–III may not be the only sources of ROS during reperfusion [41]. It is possible that during the reperfusion phase of IR injury, p66^Shc^ induces additional ROS generation via a putative direct effect on oxidoreductase activity, which depends on electron leak from upstream complexes (e.g. I and III) and then becomes an additional source of ROS production [6], [13] that may exacerbate the ROS generated from damaged complexes. This could initiate a vicious cycle of excess ROS emission by mitochondrial ROS-induced-ROS release (RIRR) during reperfusion.

### Long duration of ischemia with reperfusion is necessary for p66^Shc^ activation

We found that 5 and 10 min ischemia plus 20 min reperfusion did not increase activation of p66^Shc^, whereas 20 and 30 min ischemia with reperfusion increased p66^Shc^ activation markedly. This indicated that a longer duration of ischemia plus reperfusion was required to activate p66^Shc^. The duration of ischemia is generally linked to the extent of injury to ETC complexes [41], [42]. In rats, complex I activity decreased early in ischemia, whereas complex III damage occurred only after a longer ischemia time [41], [43]. Consistent with the time-dependent ischemic damage to ETC complexes, ROS emission also displays an ischemia duration dependent pattern. In the guinea pig heart, we showed a correlation between NADH, O_2_^−•^ levels and duration of ischemia (Fig. 4A, B) [15], [18], [24]. Our present (Fig. 4B) and past [15], [27] results in guinea pig isolated hearts showed small amounts of O_2_^−•^ were generated during early (1–20 min) global ischemia followed by a marked surge in O_2_^−•^ generation during late (25–30 min) global ischemia. Therefore, the longer durations of ischemia (20 and 30 min), when followed by at least 10 min reperfusion, are likely to have induced ETC complex damage leading to enhance ROS emission. Excess ROS (possibly H_2_O_2_), beyond a certain threshold, may act as deleterious redox-signaling molecules that lead to activation of p66^Shc^, which in turn enhances mROS generation to induce further oxidative damage to ETC complexes leading to RIRR.

Unlike 30 min ischemia, 20 min ischemia (both followed by 20 min reperfusion) induced p66^Shc^ activation (Fig. 3), but had no significant effect on cardiac function on reperfusion (data not shown). This suggests that p66^Shc^ is more sensitive to redox modulation than the measured cardiac function during IR injury. These observations show overall that p66^Shc^ participates in emitting ROS involved in cardiac dysfunction during longer ischemia times as with reperfusion.

### Amobarbital decreases activation of p66^Shc^ during cardiac IR injury

Our current and previous studies [15] show that amobarbital, when present during ischemia and early reperfusion, preserved NADH (Fig. 4A), minimized O_2_^−•^ emission (Fig. 4B), and reduced mitochondrial Ca^2+^ overload [15], which concomitantly improved functional recovery (Fig. 4 C–F) and reduced infarction when compared to IR only (control) [15]. Furthermore, in this study we also found that compared to IR alone, amobarbital reduced activation of p66^Shc^ induced by ischemia and reperfusion (Fig. 5). Since amobarbital reversibly binds at the rotenone site of complex I to attenuate electron transfer [17], [44], [45], this suggests that amobarbital, when present in the tissue during ischemia, attenuates activation of p66^Shc^ by blunting electron transfer to reduce O_2_^−•^ generation. Our observations further suggest that amobarbital better protected mitochondria and reduced myocardial injury during reperfusion, possibly due to attenuated p66^Shc^ activation and its concomitant translocation into mitochondria to modulate ROS emission.

When p66^Shc^ is activated and translocated into mitochondria, it becomes a pro-apoptotic protein that induces apoptosis by altering the redox state [6]; however, the detailed mechanism for this remains unclear [46]. Amobarbital-induced attenuation of p66^Shc^ activation during IR demonstrates that p66^Shc^ is affected by electron transfer related events that modulate the ETC. Thus, insofar as amobarbital was presumed present in the myocardium during ischemia, it may have reduced activation of p66^Shc^ during reperfusion by decreasing ischemic ROS emission. Hence, the amobarbital-induced decrease in ROS production during ischemia appears to decrease ROS emission on reperfusion, attenuate activation of cytosolic PKCβII, reduce activation and translocation of p66^Shc^ into mitochondria, and ultimately, decrease mitochondrial and cellular damage.

### PKCβ induces p66^Shc^ activation and its translocation into mitochondria during IR

The signaling pathways that lead to p66^Shc^ phosphorylation at Ser36 during cardiac ischemia and/or reperfusion have not been reported before. In this study, we show that inhibiting PKCβ with hispidin reduced both p66^Shc^ phosphorylation at Ser36 and mitochondrial translocation during IR (Fig. 6 B and C). These results indicate that p66^Shc^ activation and its eventual translocation into mitochondria during IR likely occur through PKCβ-mediated signaling pathways; this is consistent with previous studies in which different stress models were used [13], [36], [47]. Several previous reports show common p66^Shc^ phosphorylation signaling pathways, indicating an independence of the stimulus and cell type. For example, incubation of human endothelial cell with oxidized LDL led to phosphorylation [36] of p66^Shc^; in MEFs, treatment with H_2_O_2_ and UV induced phosphorylation [13] of p66^Shc^; and A549 and RAW 264.7 cells treated with Taxol, an antitumor drug, induced phosphorylation [48] of p66^Shc^. Among these stimuli and cell types, the signaling pathways that induce p66^Shc^ phosphorylation first involve activating PKCβ [13], [36], followed by activation of other kinases [36], [47], [48] that directly induce phosphorylation of p66^Shc^ at Ser36 [36].

The role of PKCβII in modulating cardiac function also has been reported. Kong et al. [14] observed significant increases in PKCβII cell membrane translocation and phosphorylation after 30 min of LAD occlusion followed by 30 min reperfusion. Moreover, PKCβ^−/−^, or its blockade with ruboxistaurin, reduced infarct size by 2.6 fold after 30 min regional ischemia and 48 h reperfusion in mice. Consistent with these findings, we found PKCβII was activated (specifically by its phosphorylation at Ser660) during IR (Fig. 6A). Furthermore, hispidin, when given before ischemia, reduced diastolic contracture (Fig. 7A) and infarct size (Fig. 7B). Although other studies also show that inhibition of PKCβ activation is protective against cardiac IR injury, our study provides evidence that PKCβII modulates cardiac IR injury by triggering the activation and translocation of p66^Shc^ into mitochondria (Fig. 6B, C).

## Summary, Conclusions and Limitations

We provide evidence that p66^Shc^ is activated and translocated from the cytosol into mitochondria in cardiac IR injury. Activation of p66^Shc^ occurred only during reperfusion via PKCβII activation. The magnitude of p66^Shc^ activation correlated with the degree of cell damage, so that the greatest activation/translocation of p66^Shc^ had the worst functional recovery, a higher incidence of VF, and a greater infarct size. Reversible blockade of complex I reduced the ROS level necessary to activate p66^Shc^. These observations imply that excess ROS and altered redox state resulting from IR injury are single or combined factors that initiate p66^Shc^ activation and mitochondrial translocation.

Persistent activation of p66^Shc^ may lead to a vicious cycle of mitochondrial RIRR as a result of redox-sensitive activation of PKCβII. Therefore, preventing p66^Shc^ activation may reduce the feed-forward cycle of RIRR and lessen reperfusion injury. The findings that p66^Shc^ mediates only detrimental ROS production during reperfusion provides a novel opportunity for pharmacological interventions that would target this redox-signaling cascade leading to p66^Shc^ activation and translocation into mitochondria after the onset of cardiac IR injury. Overall, this study provides additional valuable insights into the possible mechanisms of how modulating electron transfer along the ETC can minimize deleterious ROS emission during cardiac IR injury as a means to improve cardiac function. A potential limitation is that attenuation of p66^Shc^ translocation into mitochondria by modulating electron transfer is less pronounced than the effect on functional recovery, which indicates that other factors in addition to the effects of p66^Shc^ on translocation exert a role on ROS emission during the progression of ischemia into reperfusion.
